# Hyperglycemia promotes Snail-induced epithelial–mesenchymal transition of gastric cancer via activating ENO1 expression

**DOI:** 10.1186/s12935-019-1075-8

**Published:** 2019-12-19

**Authors:** Xin Xu, Bang Chen, Shaopu Zhu, Jiawei Zhang, Xiaobo He, Guodong Cao, Bo Chen

**Affiliations:** 1 Anhui Medical University, Hefei, 230022 China; 2 Department of General Surgery, The First Affiliated Hospital of Anhui Medical University, 218 JiXi Avenue, Hefei, 230022 Anhui China

**Keywords:** Hyperglycemia, Epithelial–mesenchymal transition, ENO1, Gastric cancer

## Abstract

**Background:**

Gastric cancer (GC) is one of the most common gastrointestinal malignancies worldwide. Emerging evidence indicates that hyperglycemia promotes tumor progression, especially the processes of migration, invasion and epithelial–mesenchymal transition (EMT). However, the underlying mechanisms of GC remain unclear.

**Method:**

Data from the Gene Expression Omnibus (GEO) and The Cancer Genome Atlas (TCGA) databases were used to detect the expression of glycolysis-related enzymes and EMT-related transcription factors. Small interfering RNA (siRNA) transfection was performed to decrease ENO1 expression. Immunohistochemistry (IHC), Western blot and qRT-PCR analyses were used to measure gene expression at the protein or mRNA level. CCK-8, wound-healing and Transwell assays were used to assess cell proliferation, migration and invasion.

**Results:**

Among the glycolysis-related genes, ENO1 was the most significantly upregulated in GC, and its overexpression was correlated with poor prognosis. Hyperglycemia enhanced GC cell proliferation, migration and invasion. ENO1 expression was also upregulated with increasing glucose concentrations. Moreover, decreased ENO1 expression partially reversed the effect of high glucose on the GC malignant phenotype. Snail-induced EMT was promoted by hyperglycemia, and suppressed by ENO1 silencing. Moreover, ENO1 knockdown inhibited the activation of transforming growth factor β (TGF-β) signaling pathway in GC.

**Conclusions:**

Our results indicated that hyperglycemia induced ENO1 expression to trigger Snail-induced EMT via the TGF-β/Smad signaling pathway in GC.

## Background

Gastric cancer (GC) is the fifth most common malignancy worldwide, and the third leading cause of cancer-related mortality [1]. It is widely known that metabolic syndrome plays an important role in GC and influences the prognosis of cancer patients [2]. Diabetes mellitus (DM) is a group of metabolic disorders characterized by persistent hyperglycemia. Cancer and DM are major public health problems worldwide, and these two conditions are closely related. A large body of epidemiologic evidence suggests that *H. pylori* infection, which is recognized as a major risk factor for GC [3], could increase the rate of DM [4, 5]. Interestingly, hyperglycemia can also increase the risk of GC posed by *H. pylori* infection [6].

Many GC patients are at an advanced stage when diagnosed and thus have a poor prognosis, and metastasis is the major reason for cancer-related death [7]. Previous studies have revealed that hyperglycemia contributes to cell invasion and metastasis in multiple cancers [8, 9]. Recent investigations have shown that epithelial–mesenchymal transition (EMT) is a reversible cellular programme, that could be a critical early event in tumor metastasis [10]. However, the mechanism of this phenomenon in GC remains unknown.

As one of the fundamental hallmarks of cancer [11], the altered energy metabolism of cancer cells has attracted increased attention. Aerobic glycolysis, known as the Warburg effect, is the most widely studied process and is characterized by increased glycolytic activity and lactate production even in the presence of adequate oxygen [12]. Tumor cells gain a steady supply of ATP and biosynthetic raw materials through aerobic glycolysis [13]. Unfortunately, hyperglycemia provides a favorable microenvironment for the growth and survival of tumor cells. The result of our bioinformatic analysis showed that among the glycolysis-related enzymes, enolase 1 (ENO1) was the most highly overexpressed gene in GC. Previous studies have demonstrated that ENO1 is deregulated in various malignancies such as glioma, hepatocellular cancer, non-small cell lung cancer and GC [14–17]. Growing evidence indicates that ENO1 plays an oncogenic role in many cancers and is associated with a poor prognosis [18]. However, data regarding the clinicopathological significance of ENO1 expression in GC tissues are limited. In addition, to the best of our knowledge, very few studies have evaluated the effect of hyperglycemia on the expression of ENO1.

In this study, we propose that hyperglycemia promotes the progression of EMT via activating ENO1 expression in GC. To test this hypothesis, the relationship between ENO1 expression and the clinicopathological features of GC patients were initially examined. Then, we detected the expression of ENO1 and EMT-related genes under different glucose concentrations. Furthermore, we investigated changes in the EMT-related genes and transforming growth factor β (TGF-β) signaling pathway expression when ENO1 was downregulated by small interfering RNA (siRNA). Here, we hope to provide theoretical and experimental support for the treatment of GC patients, especially those with DM.

## Materials and methods

### Online databases

To detect the expression level of glycolysis-related enzymes in GC, we downloaded the gene expression profiling dataset (GSE79973), which included 10 pairs of GC tissues and adjacent non-tumor mucosae, from the Gene Expression Omnibus (GEO) database (http://www.ncbi.nlm.nih.gov/geo/). The data analysis was performed by GEO2R (http://www.ncbi.nlm.nih.gov/geo/geo2r/). We also downloaded RNA-Seq data of 375 GC tissues and 32 normal tissues from The Cancer Genome Atlas (TCGA). GSE84437, which contains 433 GC tissues, was selected to investigate the relationship between ENO1 and Snail expression. Survival analysis was performed to assess whether the expression of ENO1 was correlated with GC patient outcomes based on the online database Kaplan–Meier Plotter (KM plotter, http://kmplot.com).

### Patients and tissue specimens

A total of 121 primary GC tissue samples and 30 adjacent nontumor tissues were collected from patients with pathologically and clinically confirmed at the First Affiliated Hospital of Anhui Medical University. In addition, 4 fresh primary cancer and paired adjacent normal tissue specimens were also collected. All patients had received radical gastrectomy without preoperative chemo- or radiotherapy between October 2011 and December 2012. The clinical and pathological data were summarized in detail, and the patients were staged according to the 8th edition AJCC staging system. The follow-up time ranged from 2 months to 65 months and the median time was 17 months.

### Cell culture and transfection

The human GC cell lines AGS and MGC803 were purchased from the Chinese Academy of Sciences (Shanghai, China). The cells were cultured in Dulbecco’s modified Eagle’s medium (DMEM; HyClone, Logan, UT) supplemented with 10% fetal bovine serum (FBS; Biological Industries, Israel) and antibiotics (100 U/mL penicillin, 100 μg/mL streptomycin). Cells were cultured in increasing concentrations of glucose at 5.5, 10, 15 and 25 mM to simulate a high-glucose environment. The cells at passages 8–15 were used for the subsequent experiments. All cells were maintained in a humidified incubator at 37 °C in an atmosphere of 95% air and 5% CO_2_.

SiRNA targeting ENO1 (siENO1) and negative control siRNA were designed and synthesized by Genechem Co., Ltd (Shanghai, China). Cells were transfected with siRNA using the transfection reagent Lipofectamine 2000 (Invitrogen) according to the manufacturer’s protocol. The ENO1 siRNA sequences were as follows: forward: 5′-CCCAGUGGUGUCUAUCGAATT-3′ and reverse: 5′-UUCGAUAGACACCACUGGGTT-3′.

### Immunohistochemical (IHC) staining and evaluation

The paraffin-embedded sections were deparaffinized in xylene and hydrated in serially diluted grades of ethanol. After blocking endogenous peroxidase with 3% H_2_O_2_, antigen retrieval was performed in a microwave oven using citrate buffer (pH 6.0). Sections were incubated with ENO1 antibody (1:100, Affinity Biosciences, OH, USA) overnight at 4 °C and subsequently overlaid with secondary antibody for 20 min at room temperature. Finally, a diaminobenzidine tetrahydrochloride (DAB) working solution was applied, and the slides were counterstained with hematoxylin.

The results were independently analyzed by two clinical pathologists according to a semi-quantitative method based on both the percentage of positive cells (0, no staining; 1, 0–25%; 2, 26%–50%; 3, 51%–75%; 4, 76%–100%) and staining intensity (0, negative; 1, weak; 2, moderate; 3, strong) [19]. The final score was the product of the staining intensity and the percentage of positive cells. High expression was defined as a scoring index ≥ 8, and low expression was defined as a scoring index of 0–8.

### RNA extraction and quantitative real-time PCR (qRT-PCR)

Total RNA was extracted from cells using TRIzol reagent (Invitrogen, USA) according to the manufacturer’s instructions. RNA was reverse-transcribed into cDNA with PrimeScript RT-polymerase (Takara Bio, Dalian, China). Then, qRT-PCR was performed with SYBR Green assay kit (Takara, Japan). The reaction conditions were as follows: initial denaturation at 95 °C for 10 min, followed by 40 cycles of denaturation at 95 °C for 20 s, annealing at 60 °C for 20 s and extension at 72 °C for 30 s. The primer sequences were as follows: ENO1 forward: 5ʹ-CCCAGUGGUGUCUAUCGAATT-3ʹ, reverse: 5ʹ-UUCGAUAGACACCACUGGGTT-3; GAPDH forward: 5′-GCATCCTGGGCTACACT-3′, reverse: 5′-CACCACCCTGTTGCTGT-3′. The 2^−ΔΔCt^ method was used to calculate the relative ENO1 mRNA expression and GAPDH was used as the internal control.

### Western blot

RIPA lysis buffer (Beyotime, Shanghai, China) was used to extract total protein. The protein concentration was quantified using a BCA protein assay kit (Beyotime, Shanghai, China). The equal quantities of proteins were separated by 6–10% SDS-PAGE gel electrophoresis and then transferred onto polyvinylidene fluoride membranes. After the membranes were blocked in 5% skim milk powder with 0.1% Tween-20 for 1 h at room temperature, they were incubated with primary antibodies against ENO1 (1:1000, Affinity Biosciences, OH, USA), E-cadherin (1:1000, Cell Signaling Technology, MA, USA), N-cadherin (1:1000, Cell Signaling Technology, MA, USA), Vimentin (1:1000, Cell Signaling Technology, MA, USA), Snail (1:1000, Elabscience Biotechnology, Wuhan, China), p-Smad2 (1:1000, Cell Signaling Technology, MA, USA), Smad2 (1:1000, Cell Signaling Technology, MA, USA), p-Smad3 (1:1000, Cell Signaling Technology, MA, USA), Smad3 (1:1000, Cell Signaling Technology, MA, USA) and TGF-β (1:1000, Cell Signaling Technology, MA, USA) overnight at 4 °C. After washing three times in TBST, the membranes were incubated with secondary antibody for 1 h at room temperature. Finally, the protein bands were visualized using an enhanced chemiluminescence (ECL) detection system. The protein band intensities were normalized to the GAPDH intensity.

### Cell counting kit-8 (CCK-8) assay

GC cell proliferation was measured with a CCK-8 assay (Dojindo, Tokyo, Japan). Briefly, cells in the logarithmic phase were plated onto 96-well plates at a density of 3000 cells per well. A volume of 10 µL of CCK-8 solution was added to each well at the indicated times (24, 48, 72, and 96 h), followed by 1.5 h of incubation. The relative optical density (OD) was measured at 450 nm using an automated plate reader (Bio-Rad, USA).

### Wound-healing assay

Differently treated cells were seeded in a 6-well plate and grown to 90% confluence in 2 mL of culture medium and incubated at 37 °C with 5% CO_2_. A 200 µL plastic tip was used to create an artificial wound. After washing with phosphate buffer saline (PBS), cells were incubated in fresh medium with 1% FBS. Images were taken at 0 and 48 h after scratching. Cell mobility = (0 h width − 48 h width)/0 h width × 100%.

### Migration and invasion assays

Cell migration and invasion assays (Corning Life Sciences, Bedford, MA, USA) were performed using 24-well plates with a pore size of 8 μm. Matrigel invasion was used to assess the GC cell migratory and invasive abilities. For migration assays, GC cells were seeded in the upper chamber with 200 µL serum-free DMEM at a density of 5 × 10^4^ cells/well and the lower chamber was filled with 600 µL of culture medium containing 20% FBS. After incubation for 24 h, the non-migrated cells were carefully removed with a wet cotton swab. Finally, the cells were stained with 4% paraformaldehyde, stained with 0.5% crystal violet and counted under a microscope (100× magnification). The cell invasion assay was carried out similarly, but the chambers were coated with Matrigel (BD Biosciences, USA) before cells were seeded on the membrane.

### Statistical analysis

The statistical analyses were performed using SPSS 19.0 (SPSS Inc., USA) and graphed using GraphPad Prism 6 (GraphPad Software Inc., USA). The data are shown as the mean ± standard deviation (SD) and were compared by Student’s *t*-test or one-way ANOVA. Associations between pathological variables were examined using Pearson’s Chi-squared test. Survival curves were generated by the Kaplan–Meier method, with statistical significance evaluated by the log-rank test. Univariate and multivariate analyses were performed by using a Cox proportional hazards model. Spearman’s correlation analysis was used to identify the correlation between ENO1 expression and EMT-related transcription factors. *P* < 0.05 was considered significant.

## Results

### ENO1 was upregulated in GC tissues and correlated with poor prognosis

**Fig. 1:**
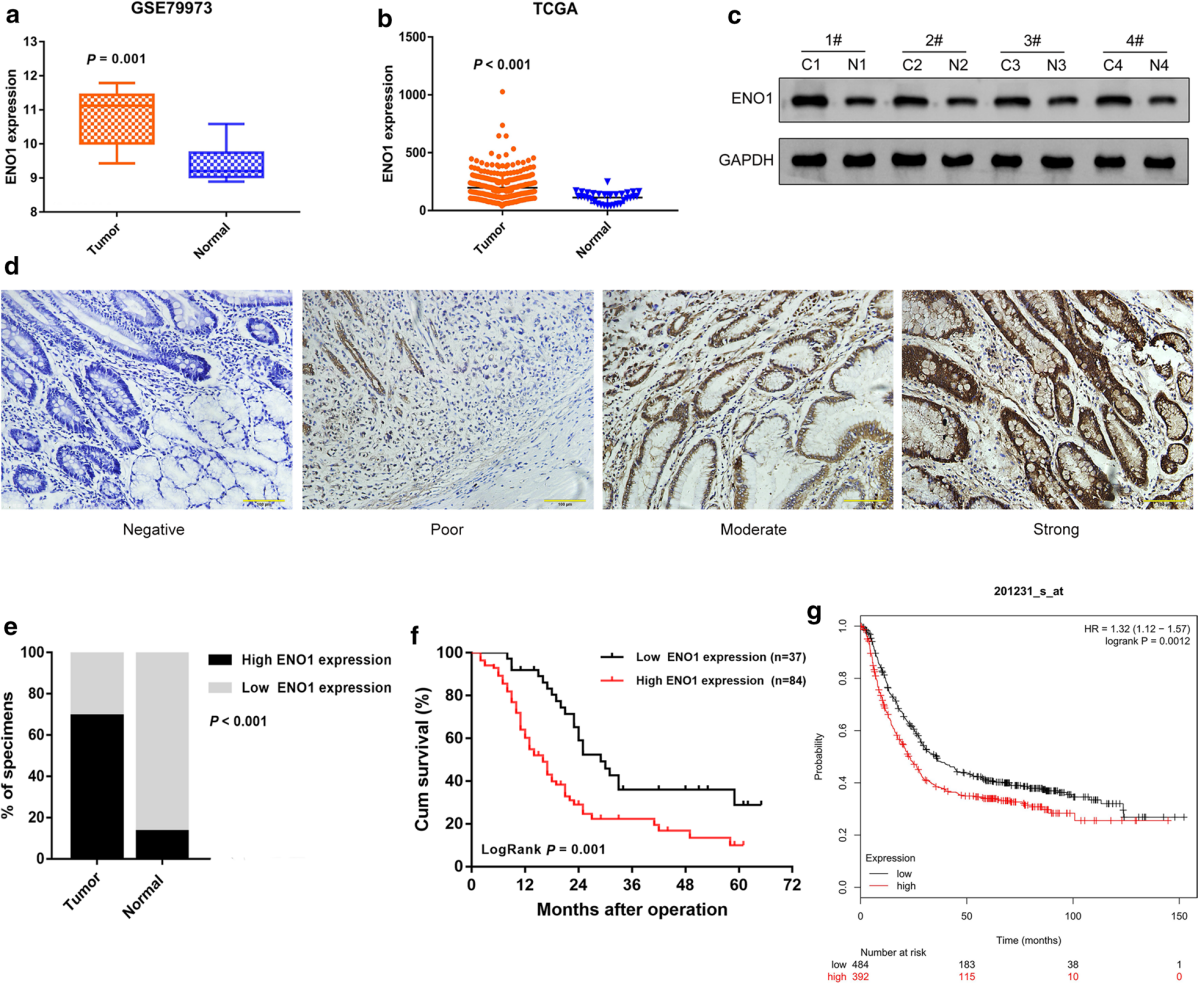
ENO1 was upregulated in GC tissue and was associated with poor prognosis. **a**, **b** The expression of ENO1 was determined based on the GSE79973 and TCGA databases. **c** Western blot analysis detected ENO1 expression in GC and adjacent normal tissues. **d** Representative ENO1 IHC staining in GC specimens (×200 magnification). **e** The statistical analysis of ENO1 expression in GC and adjacent normal tissues. **f**, **g** Kaplan–Meier survival curves of GC patients with low and high ENO1 expression in our study or in the KM plotter database. *P < 0.05

To identify the potential enzymes involved in GC progression, the mRNA expression data of glycolysis-related genes were extracted from the GEO database (GSE79973) for bioinformatic analysis. As shown in Table 1, GC tissues exhibited elevated expression of ENO1 (adjusted *P* value = 0.009) and PKM (adjusted *P* value = 0.039). As the most significantly upregulated glycolysis-related gene, ENO1 was chosen for further study. The results based on GSE79973 and TCGA data analysis showed that the expression of ENO1 was significantly higher in tumor tissues compared with that in normal tissues (Fig. 1a, b). A similar result was found with Western blotting (Fig. 1c).

**Table 1:** The detail information of glycolysis-related genes expression based on GSE79973

ID	Gene symbol	adj. *P*-value^a^	*P*-value	logFC^b^
217294_s_at	ENO1	0.008592	8.94E−05	1.4491932
201251_at	PKM	0.038831	1.96E−03	1.1217714
227068_at	PGK1	0.065488	5.55E−03	0.5725665
200650_s_at	LDHA	0.101375	1.29E−02	0.4574512
201037_at	PFKP	0.20671	4.89E−02	0.4500467
M33197_5_at	GAPDH	0.120333	1.80E−02	0.4460345
205736_at	PGAM2	0.662796	4.22E−01	0.3678693
210976_s_at	PFKM	0.498706	2.48E−01	0.2746726
201313_at	ENO2	0.617634	3.70E−01	0.2600634
211167_s_at	GCK	0.821298	6.49E−01	0.192802
200886_s_at	PGAM1	0.80052	6.14E−01	0.0622324
214687_x_at	ALDOA	0.243907	6.56E−02	− 0.2745198
201102_s_at	PFKL	0.154507	2.86E−02	− 0.3557137
201249_at	SLC2A1	0.52358	2.70E−01	− 0.3966075
217009_at	PGK2	0.581161	3.29E−01	− 0.4859326
202934_at	HK2	0.435741	1.92E−01	− 0.5911105
201030_x_at	LDHB	0.273184	8.04E−02	− 0.708974
1553837_at	PGAM5	0.060965	4.86E−03	− 0.8898033
206603_at	GLUT4	0.198491	4.53E−02	− 1.3422707
209696_at	FBP1	0.018156	4.22E−04	− 1.3654888
206844_at	FBP2	0.04158	2.29E−03	− 4.0148564
204704_s_at	ALDOB	0.009369	1.12E−04	− 4.5435921

^a^Adjusted *P*-value

^b^Log fold change

Furthermore, to determine the clinical significance of ENO1, we used IHC staining to examine the ENO1 protein expression in GC tissues. High ENO1 protein expression was present in 84/121 (69.4%) GC specimens, while 26/30 (86.7%) adjacent normal tissues showed low expression (*P* < 0.001, Table 2). Representative images with different levels of ENO1 and statistical analysis are shown in Fig. 1d, e. The results of the clinicopathological correlation study revealed that ENO1 overexpression was significantly associated with lymph node metastasis (*P* = 0.003), distant metastasis (*P* = 0.015) and TNM stage (*P* < 0.001) (Table 3). Notably, we observed that patients with high preoperative blood glucose had slightly increased levels of ENO1 in the GC tissues (*P* = 0.039). Overall survival (OS) analysis indicated that the patients with high expression of ENO1 had a shorter survival time than those with low expression (logrank *P *= 0.001, Fig. 1f). As shown in Fig. 1g, the result was further verified based on KM plotter. In addition, univariate Cox regression analysis showed that preoperative blood glucose (*P* < 0.001), lymph node metastasis (*P* = 0.013), distant metastasis (*P* < 0.001), TNM stage (*P* = 0.006) and ENO1 expression (*P* = 0.001) were associated with OS. Multivariate Cox regression analysis showed that preoperative blood glucose (*P* = 0.031), distant metastasis (*P* = 0.003) and ENO1 expression (*P* = 0.024) were independent prognostic factors for OS of GC (Table 4).

**Table 2:** The expression of ENO1 in GC and adjacent normal tissues

Parameters	Cases	ENO1 expression		χ^2^	*P*-value
		High	Low		
GC tissues	121	84	37	31.103	< 0.001*
Adjacent normal tissue	30	4	26		

* Statistically significant (*P* < 0.05)

**Table 3:** Correlation of ENO1 expression with clinicopathologic parameters in GC patients

Parameters	Cases	ENO1 expression		χ^2^	*P*-value
		High	Low		
Gender				1.422	0.233
Male	75	55	20		
Female	46	29	17		
Age (years)				0.124	0.724
≥ 60	94	66	28		
< 60	27	18	9		
Blood glucose (mmol/L)^a^				4.269	0.039*
≥ 7	19	17	2		
< 7	102	67	35		
Tumor size (cm)				0.230	0.631
≥ 5	68	46	22		
< 5	53	38	15		
Differentiation				0.767	0.381
Well + moderate	39	25	14		
Poor	82	59	23		
Lymph node metastasis				8.687	0.003*
Yes	90	69	21		
No	31	15	16		
Distant metastasis				5.868	0.015*
Yes	12	12	0		
No	109	72	37		
TNM stage				23.869	< 0.001*
I + II	43	18	25		
III + IV	78	66	12		

^a^Preoperative fasting blood glucose

* Statistically significant (*P* < 0.05)

**Table 4:** Univariate and multivariate analysis of clinicopathological variables and ENO1 expression associated with overall survival

Parameters	Univariate analysis		Multivariate analysis	
	HR (95% CI)	*P*-value	HR (95% CI)	*P*-value
Gender	1.075 (0.688–1.679)	0.750		
Age (years)	1.033 (0.611–1.747)	0.903		
Blood glucose (mmol/L)^a^	3.082 (1.708–5.558)	< 0.001*	2.020 (1.067–3.825)	0.031*
Tumor size (cm)	1.329 (0.852–2.071)	0.209		
Differentiation	1.624 (1.000–2.639)	0.050		
Lymph node metastasis	1.983 (1.159–3.394)	0.013*	1.447 (0.646–3.242)	0.369
Distant metastasis	5.164 (2.588–10.307)	< 0.001*	3.063 (1.461–6.424)	0.003*
TNM stage	1.926 (1.207–3.072)	0.006*	1.067 (0.511–2.224)	0.864
ENO1 expression	2.310 (1.404–3.800)	0.001*	1.867 (1.086–3.209)	0.024*

^a^Preoperative fasting blood glucose

* Statistically significant (*P* < 0.05)

### Hyperglycemia facilitated GC cell proliferation, migration, invasion and ENO1 expression

**Fig. 2:**
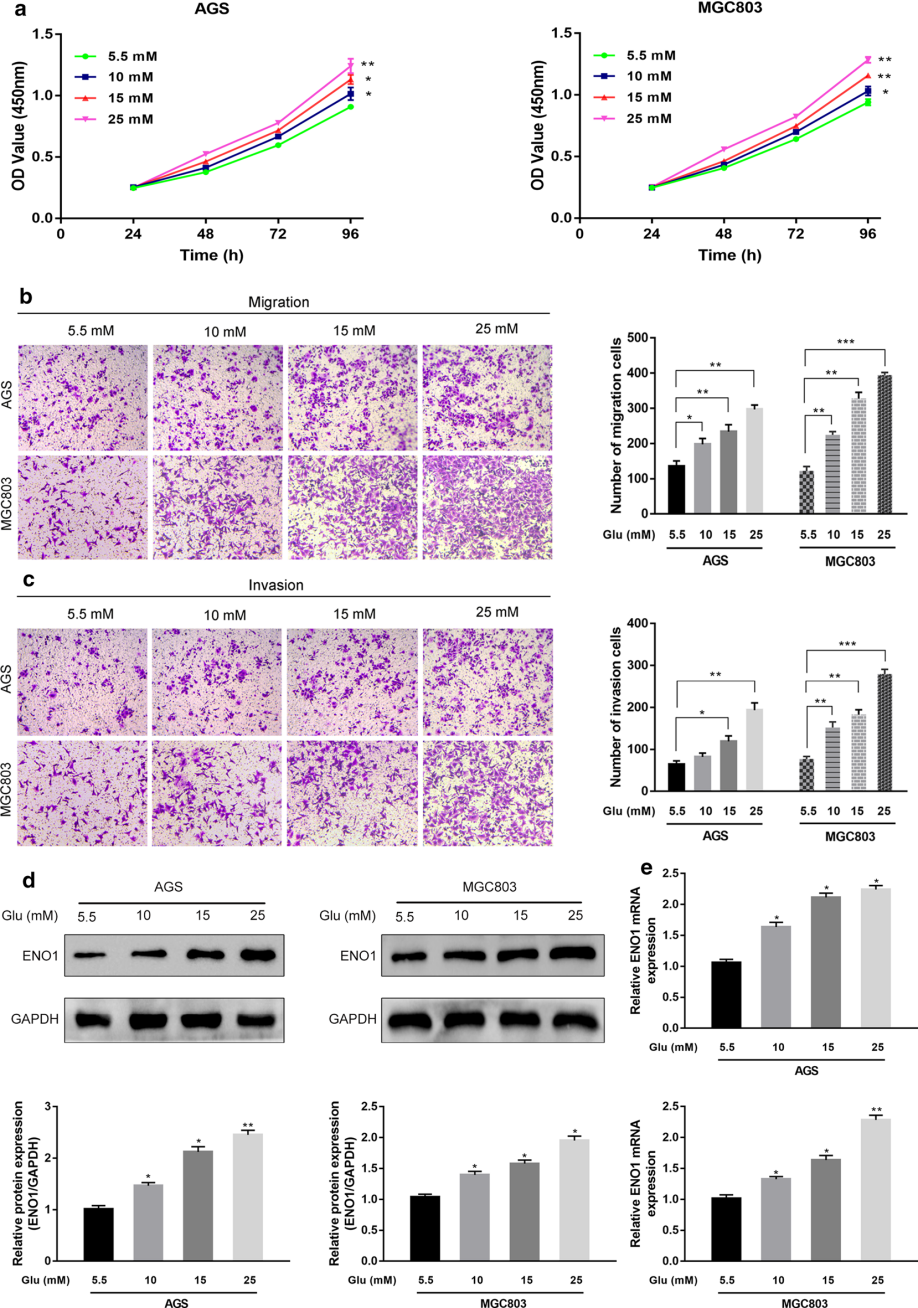
Hyperglycemia promoted GC cell proliferation, migration, invasion and ENO1 expression. **a** Cell proliferation was detected by CCK-8 assays in AGS and MGC803 cells incubated with different glucose concentrations for 24, 48, 72 and 96 h. **b**, **c** The effect of hyperglycemia on GC cell migration and invasion was determined by transwell migration and invasion assays. **d**, **e** Western blot analysis and qRT-PCR were used to examine ENO1 expression under different glucose concentrations in GC. *P < 0.05, **P < 0.01, ***P < 0.001

As we observed that high blood glucose was associated with poor prognosis, we treated GC cells (AGS and MGC803) in culture with different concentrations of glucose (5.5, 10, 15 and 25 mM) in vitro. Subsequently, to investigate the effect of high glucose on the proliferation, migration and invasion of GC cells, CCK-8, migration and invasion assays were performed, respectively. As shown in Fig. 2a, compared with that in the normal glucose group, the proliferation rate in all hyperglycemia groups was significantly upregulated in the two cell lines at various time points. The results of transwell assays revealed that the migratory and invasive capabilities of the GC cells were significantly enhanced in the hyperglycemia groups (Fig. 2b, c). In addition, as the concentration of glucose increased, the ENO1 mRNA and protein levels gradually increased in the AGS and MGC803 cells (Fig. 2d, e). In other words, the expression of ENO1 appeared to be induced by glucose in a concentration-dependent manner in vitro. Together, our present findings indicated that hyperglycemia promoted GC cell proliferation, migration and invasion, as well as ENO1 expression.

### ENO1 silencing suppressed hyperglycemia-induced proliferation, migration and invasion of GC cells

**Fig. 3:**
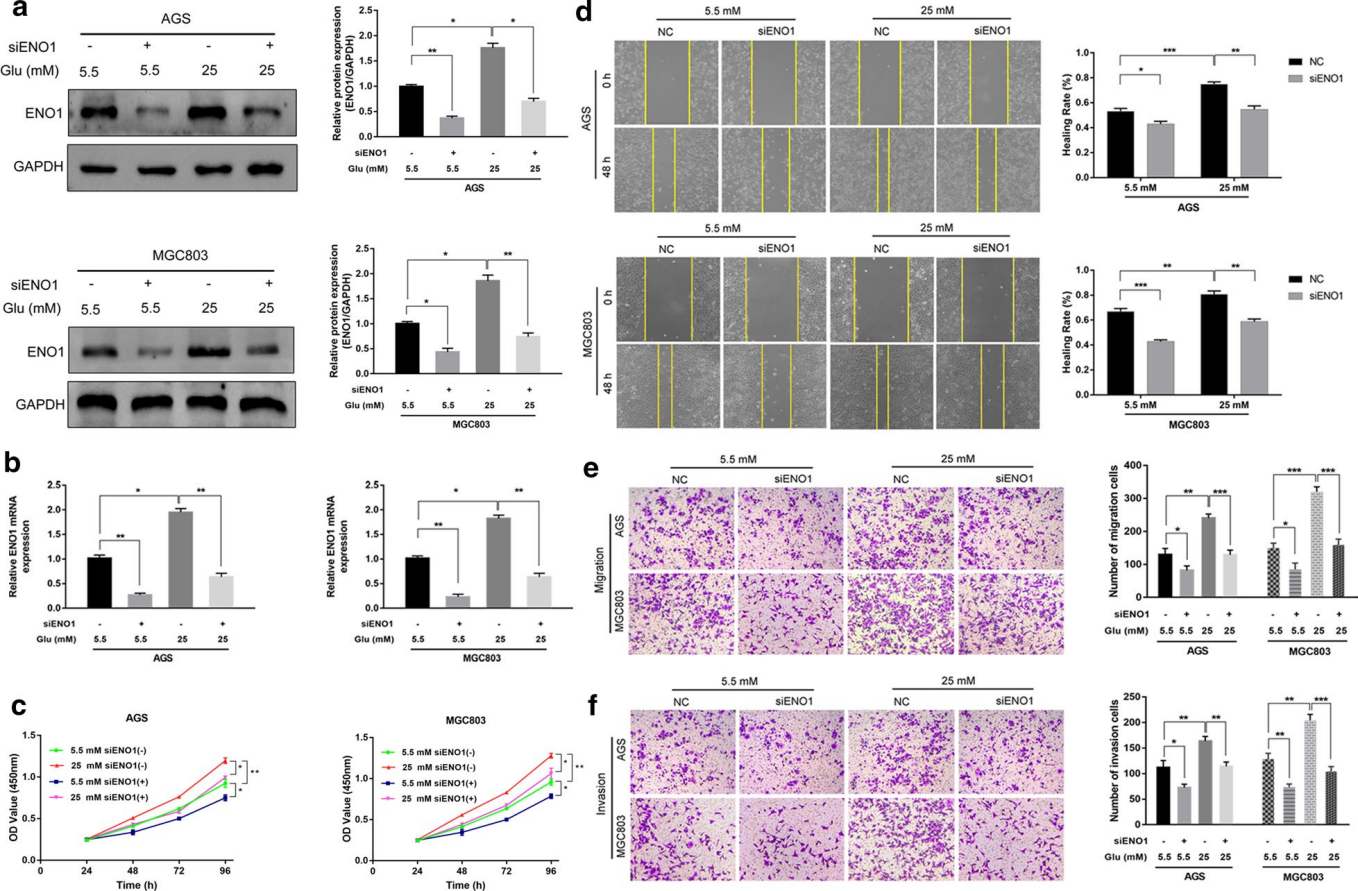
Knockdown of ENO1 suppressed the hyperglycemia-induced GC malignant phenotype in AGS and MGC803 cells. **a**, **b** Western blot analysis and qRT-PCR were performed to verify the transfection efficiency of ENO1. **c** CCK-8 assays were used to evaluate the effect of ENO1 knockdown on cell proliferation. **d**, **e** The effect of ENO1 silencing on migration was examined by wound healing and transwell migration assays. **f** Transwell invasion assays were used to assess the invasive ability of GC cells transfected with siENO1 in the normal and high glucose groups. *P < 0.05, **P < 0.01, ***P < 0.001

To further elucidate the role of ENO1, we first downregulated ENO1 expression using siRNA in AGS and MGC803 cells, which were cultured with 5.5 or 25 mM glucose. Western blot and qRT-PCR analysis showed that the expression of ENO1 was significantly decreased by siRNA (Fig. 3a, b). ENO1 knockdown significantly weakened the GC cell proliferative capacity in both the normal glucose and hyperglycemia groups compared with the control groups (Fig. 3c). Next, wound healing and transwell assays were employed to further investigate the effects of ENO1 on the migration and invasion of GC cell in vitro. The results of the wound healing and transwell migration assays demonstrated that GC cell migration was effectively inhibited by ENO1 silencing (Fig. 3d, e). Consistently, the transwell invasion assay revealed a similar effect on AGS and MGC803 cells invasion with different glucose concentrations (Fig. 3f). These results indicated that ENO1 silencing could inhibit hyperglycemia-induced GC cell growth, migration and invasion.

### Snail‐mediated EMT was responsible for hyperglycemia/ENO1‐induced GC malignant phenotype

**Fig. 4:**
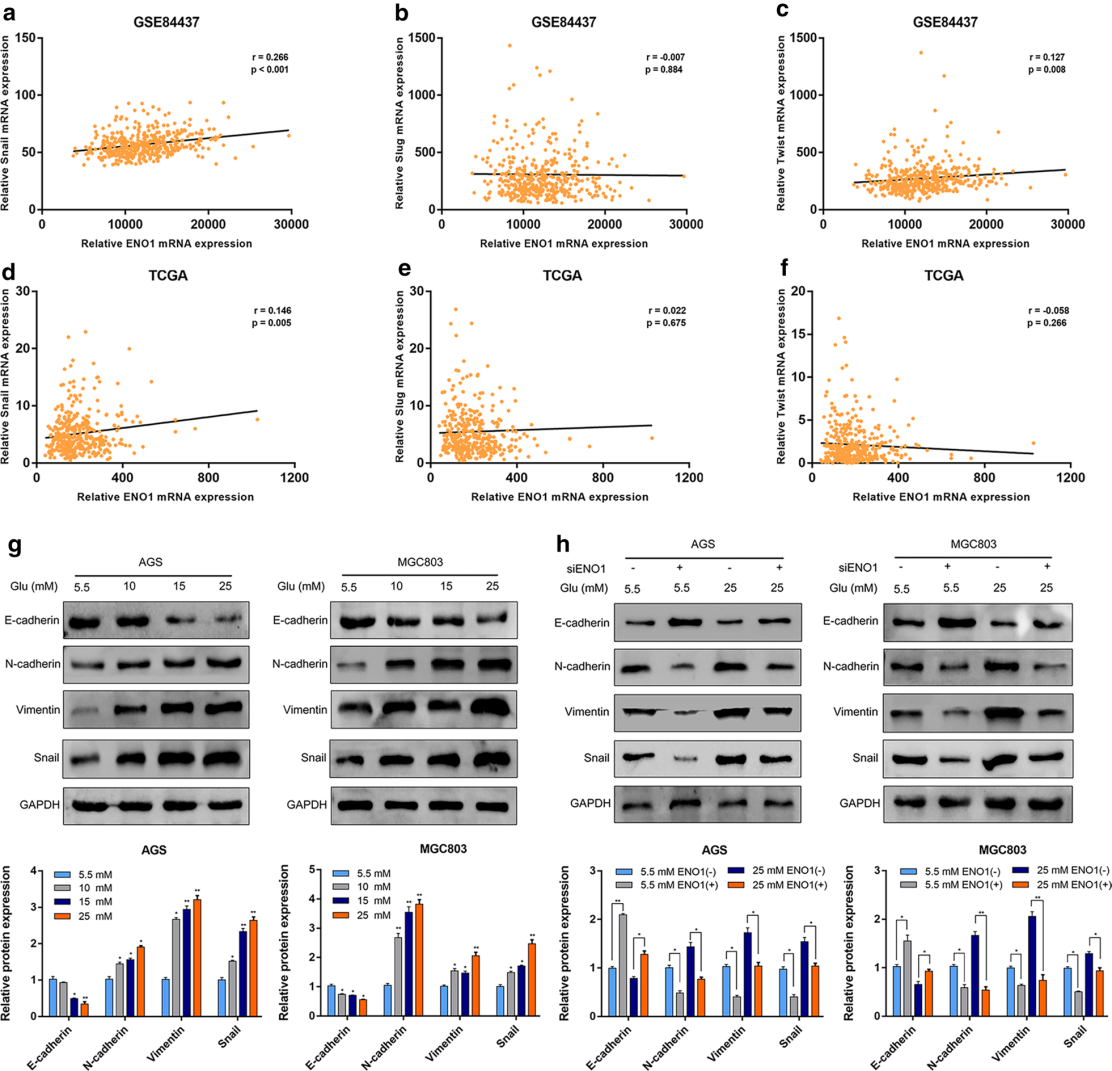
Knockdown of ENO1 could partly reverse hyperglycemia-induced EMT in AGS and MGC803 cells. **a**–**c** The relationship between the expression of ENO1 and EMT-related transcription factors (Snail, Slug and Twist) was examined by correlation analysis based on GSE84437. **d**–**f** The relationship between the expression of ENO1 and EMT-related transcription factors (Snail, Slug and Twist) was examined by correlation analysis based on TCGA database. **g** Western blot analysis was performed to measure the expression of EMT-related markers under different glucose concentrations. **h** The effect of ENO1 knockdown on EMT-related marker expression was shown by Western blot. *P < 0.05, **P < 0.01

It is well known that the EMT can enhance many cancer metastatic phenotypes, including migration, invasion and metastasis, during tumor progression. The activation of EMT is regulated by multiple transcription factors, such as Snail, Slug and Twist. To explore the possible relationship between ENO1 and EMT-related transcription factors, we performed correlation analysis using data from a large sample from GSE84437 (including 433 GC samples) and TCGA (including 375 GC samples). Among these transcription factors, only Snail was significantly positively correlated with the expression of ENO1 in both the GSE84437 (r = 0.266, *P* < 0.001) and TCGA (r = 0.146, *P* = 0.005) datasets (Fig. 4a–f).

As shown in Fig. 4g, the expression of E-cadherin (epithelial marker) was decreased, while the expression of N-cadherin and Vimentin (mesenchymal markers) was increased with increasing glucose concentrations. Snail expression was also significantly upregulated in the hyperglycemia groups compared with the normal glucose group. This result indicated that a hyperglycemic environment might induce GC cell EMT and stimulate migration and invasion in vitro. Mechanistically, the Western blot results analysis showed that ENO1 knockdown suppressed hyperglycemia-induced decreases in E-cadherin and increases in N-cadherin, Vimentin and Snail (Fig. 4h). These data revealed that ENO1 might play an important role in hyperglycemia-induced EMT.

### Downregulation of ENO1 inhibited the TGF-β/Smad signaling pathway

**Fig. 5:**
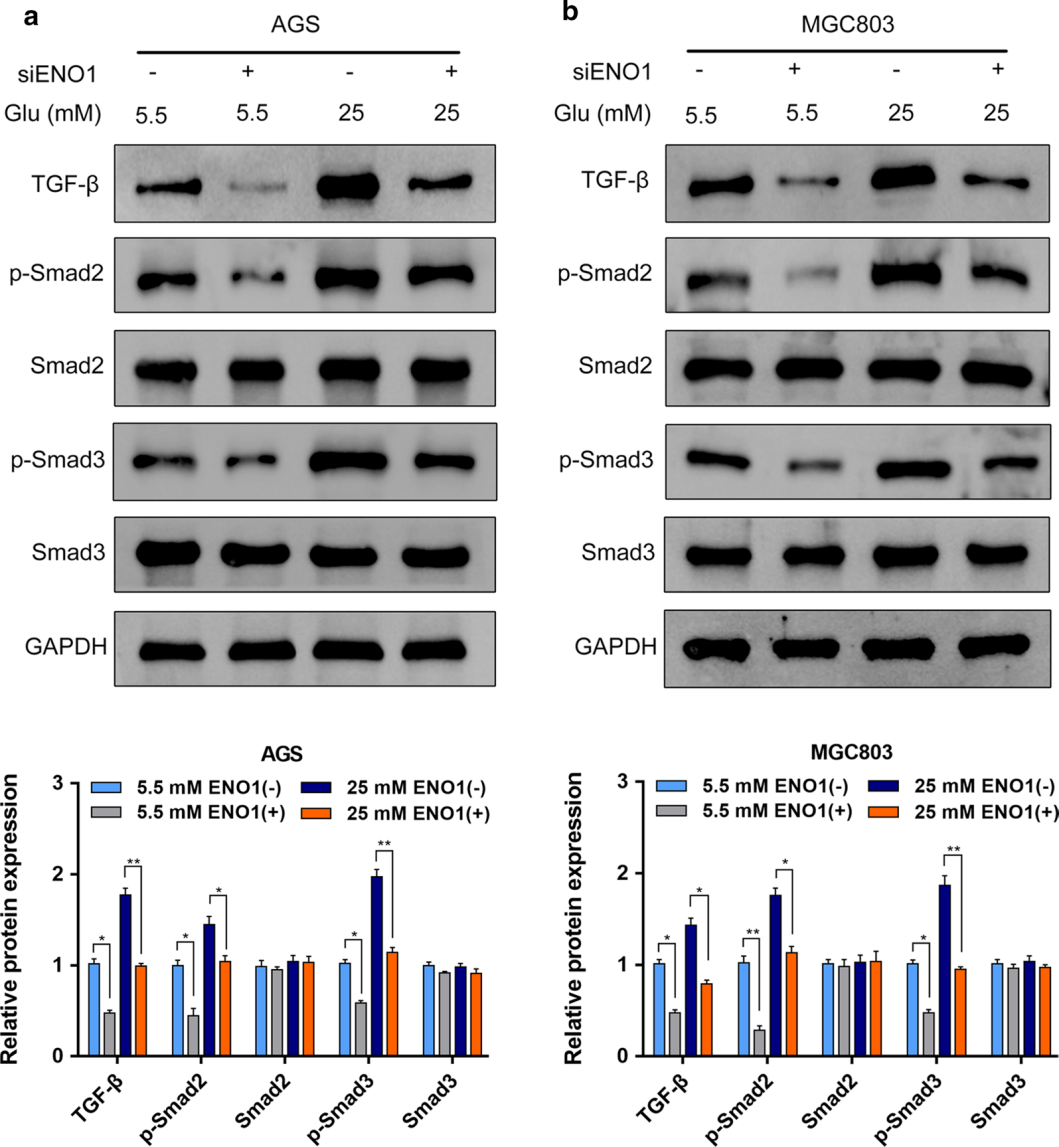
Knockdown of ENO1 inhibited the TGF-β/Smad signaling pathway in AGS and MGC803 cells. **a**, **b** Western blot analysis was performed to measure the expression levels of TGF-β, Smad2, p-Smad2, Smad3 and p-Smad3 under different glucose concentrations. *P < 0.05, **P < 0.01

The TGF-β/Smad signaling pathway has a critical role in cancer development, and more importantly, it could also regulate EMT to promote cancer cell migration, invasion and metastasis. To elucidate the specific mechanisms, we further examined the effect of ENO1 knockdown on the activation of the TGF-β/Smad signaling pathway in AGS and MGC803 cells. Our results revealed that the expression levels of TGF-β, phosphorylated-Smad2 (p-Smad2) and phosphorylated-Smad3 (p-Smad3) were decreased with ENO1 knockdown, whereas the total Smad2 and Smad3 expression levels were not significantly different between the normal glucose and hyperglycemia groups (Fig. 5a, b). Collectively, these data implied that hyperglycemia/ENO1 induced GC malignant phenotype at least partially by modulating the TGF-β/Smad signaling pathway.

## Discussion

Despite recent developments in diagnosis and treatment, the prognosis of GC patients remains unfavorable mainly due to recurrence and distant metastasis [20]. Accumulating data and studies have shown that tumor patients with hyperglycemia, including those with GC, always have poor prognosis [9, 21–23]. Surprisingly, postoperative hyperglycemia was associated with poor outcomes even in non-diabetic patients undergoing elective gastric surgery for cancer [24]. Hyperglycemia could enhance the proliferative capacity of non-tumorigenic and malignant mammary epithelial cells, and increase the risk of breast cancer in premalignant lesions [25]. In a recent study, hyperglycemia negatively regulated the killing effects of NK cells to achieve immune escape in pancreatic cancer [26]. Additionally, GC cells tend to show multidrug resistance and reduced susceptibility to chemotherapy drugs under high glucose conditions [27]. Another study reported that hyperglycemia can enhance oxaliplatin chemoresistance and lead to poor clinical outcomes in stage III colorectal cancer patients receiving adjuvant chemotherapy [28]. This study aimed to investigate the effects of hyperglycemia on the GC malignant phenotype and the underlying mechanisms. The results demonstrated that high glucose could enhance the malignant phenotype including increased GC cell proliferation, migration and invasion. Moreover, high glucose upregulated the expression of ENO1. Notably, knockdown of ENO1 could significantly reverse the hyperglycemia-induced GC malignant phenotype. Mechanistically, further research revealed that Snail‐mediated EMT played a vital role in the hyperglycemia/ENO1‐induced GC malignant phenotype.

Hyperglycemia is essential for the initiation and progression of carcinogenesis. High glucose provides sufficient energy and creates a favorable microenvironment for tumor cells. Many studies have shown that hyperglycemia stimulates tumor cell glycolysis by regulating the expression levels of glycolytic enzymes. For example, a new study reported that hyperglycemia could enhance glycolysis by increasing LDHA activity and HK2, PFKP expression to promote pancreatic cancer progression [29]. Increased expression of LDHA was also detected in the colorectal epithelium of patients with DM, which suggested increased aerobic glycolysis [30]. ENO1, one of the key enzymes in the glycolytic process, catalyses the formation of phosphoenolpyruvate from 2-phosphoglycerate [31]. In fact, ENO1 is a multifunctional protein. In addition to its catalytic function, ENO1 has non-glycolytic functions, such as cell surface plasminogen binding, maintenance of mitochondrial membrane stability, transcriptional repressor activity in the nucleus, as well as chaperon and vacuole fusion activity in the cytoplasm [32]. Our results revealed that ENO1 was highly expressed and played an important role in GC development, which was consistent with previous studies [17, 33, 34]. Chen et al. [35] reported that the expression of ENO1 could be upregulated by *H. pylori* infection and the bacterial oncoprotein CagA, thereby enhancing the risk for GC. We observed, possibly for the first time, that the expression of ENO1 was significantly higher in the hyperglycemia groups than the normal glucose group. This finding indicated that a high-glucose environment can also enhance the glycolysis level of GC cells. ENOblock, a small molecule nonsubstrate analogue that inhibits ENO1, was shown to suppress colon cancer cell metastasis and induce cellular glucose uptake [36]. A recent study demonstrated that treatment with ENOblock could inhibit gluconeogenesis, adiposity and obesity-related inflammation [37]. Furthermore, it has been proved that ENOblock could reduce hyperglycemia and hyperlipidemia and decrease secondary diabetic complications in a mammalian model of type 2 DM [38]. Therefore, targeted inhibition of ENO1 in patients with GC and DM may yield unexpected results. We believe that relevant clinical trials will be possible in the near future.

There is no doubt that EMT plays a vital role in tumor invasion and metastasis. During EMT, epithelial cells lose their apical-basal polarity, reorganize the cytoskeleton, show increased cell motility and gain mesenchymal morphology [39]. Notably, high glucose concentrations modulate EMT-related protein expression and morphology to enhance cell migration and invasion in several cancers [40–42]. Interestingly, ENO1 has recently been found to modulate EMT progression [14, 16, 17]. Additionally, there was evidence that surface ENO1 was shown to exert its previously mentioned non-glycolytic effects to induce pericellular plasminogen activation, promote extracellular matrix degradation and increase invasion and metastasis of tumor cells [43]. TGF-β is a multifunctional cytokine that is involved in cancer progression, including EMT, immune evasion, metastasis and chemotherapy resistance [44]. High glucose could also induce nuclear translocation of Smad3 and enhance the activation of TGF-β/Smad signaling pathway [45]. In our study, we found that hyperglycemia promoted GC cell proliferation, migration, invasion and EMT, as well as ENO1 expression. Data from online databases showed the expression of Snail was significantly positively correlated with ENO1 expression. Furthermore, our results demonstrated that downregulation of ENO1 could partially reverse the above effects of hyperglycemia. Further research confirmed that ENO1 knockdown significantly inhibited the TGF-β/Smad signaling pathway in both the normal glucose and hyperglycemia groups. All the results were consistent with our hypothesis that hyperglycemia-induced ENO1 overexpression promotes a malignant phenotype in GC via Snail-induced EMT through the TGF-β/Smad signaling pathway.

However, our study has some limitations that must be considered. First, GC cells cultured with different concentrations of glucose cannot represent the in vivo system. Second, we did not investigate the effect of hyperglycemia on cellular glycolytic activity. Third, an animal model and further research on the relationship between ENO1 and Snail may also be needed.

## Conclusion

In conclusion, our results indicate that overexpression of ENO1 is observed in GC and is associated with the clinicopathological features and prognosis of patients, suggesting that ENO1 might be a prognostic indicator in GC. High glucose levels can upregulate ENO1 expression, which stimulates Snail-induced EMT through the TGF-β/Smad signaling pathway in GC. We strongly propose perioperative glycemic control as a potential strategy for clinical management of GC.

## Data Availability

Not applicable.

## Abbreviations

GC: gastric cancer
ENO1: enolase 1
EMT: epithelial–mesenchymal transition
GEO: Gene Expression Omnibus
TCGA: The Cancer Genome Atlas
TGF-β: transforming growth factor β
siRNA: small interfering RNA
IHC: immunohistochemical
DM: diabetes mellitus
qRT-PCR: RNA extraction and quantitative real-time PCR
OS: overall survival
